# Supplementary material for: First report of a major management target species, chironomid Paratanytarsus grimmii (Diptera: Chironomidae) larvae, in drinking water treatment plants (DWTPs) in South Korea
Source: PLoS One. 2025 Jan 10;20(1):e0315390. doi: 10.1371/journal.pone.0315390 (PMC11723544; doi:10.1371/journal.pone.0315390)
Supplement: Attachment — Submitted filename: 241120_Response to reviewer.docx [file pone.0315390.s002.docx]

20^st^ November, 2024

Editors

PLOS ONE

Dear Editors:

We are sending the revised manuscript titled “First report of a major management target species, chironomid *Paratanytarsus grimmii* (Diptera: Chironomidae) larvae, in drinking water treatment plants (DWTPs) in South Korea” for consideration to publish in your prestige journal.

Ensuring the supply of safe and high-quality drinking water can be compromised by the presence of chironomid larvae in drinking water treatment plants (DWTPs), which may contaminate municipal water systems through freshwater resources. Chironomids are dominant species known for their resilience to a broad range of extreme aquatic environments. This study aimed to identify the morphological characteristics and obtain genetic information of the chironomid *Paratanytarsus grimmii* found in the water intake source and freshwater resource of DWTPs in Korea, highlighting the potential possibility of a parthenogenetic chironomid outbreak within DWTP networks. The distribution of chironomid larvae at the water intake source site (DY) of the Danyang DWTP and the freshwater resource (ND) of the Nakdong River was investigated. A total of 180 chironomid individuals, encompassing three subfamilies and six species from six 6 genera were identified at the DY site, with *Procladius nigriventris* being the dominant species. At the ND site, fifty chironomid individuals, encompassing two subfamilies and six species from six genera, were identified, with *Cricotopus sylvestris* being the dominant species. The morphological characteristics of the head capsule, mentum, mandible, and antennae of six *P. grimmii* larvae collected from the DY and ND sites were characterized. DNA barcoding and phylogenetic analysis revealed distinct mitochondrial diversities between the *P. grimmii* larvae from DY and those from ND. These results provide crucial information for the morphological identification and DNA barcoding of the key management target chironomid *P. grimmii* larvae, which can be used to detect the occurrence of this chironomid species in DWTPs.

We believe that our findings would satisfy your journal requirements for the publication of this paper. Therefore, please be kind enough to evaluate our manuscript and inform us your comments. The English in the manuscript has been corrected by a professional science-editing service (EssayReview). In addition, our specific responses to the points raised by the reviewers are presented below.

**Reviewers' comments:**

Reviewer's Responses to Questions

**Comments to the Author**

1. Is the manuscript technically sound, and do the data support the conclusions?

The manuscript must describe a technically sound piece of scientific research with data that supports the conclusions. Experiments must have been conducted rigorously, with appropriate controls, replication, and sample sizes. The conclusions must be drawn appropriately based on the data presented.

Reviewer #1: Yes

 🡪 Thank you for the comment of reviewer #1.

2. Has the statistical analysis been performed appropriately and rigorously?

Reviewer #1: Yes

 🡪 Thank you for the comment of reviewer #1.

3. Have the authors made all data underlying the findings in their manuscript fully available?

The [PLOS Data policy](http://track.editorialmanager.com/CL0/http:%2F%2Fwww.plosone.org%2Fstatic%2Fpolicies.action%23sharing/1/010f0192c49e8dc7-30b1bdc1-4be1-4abe-9718-1af2ec0c4ea7-000000/NjGC1dbhEA6Uzm3sgPEjSwJ9s9fNHrpgbj5VMylfTH0=182) requires authors to make all data underlying the findings described in their manuscript fully available without restriction, with rare exception (please refer to the Data Availability Statement in the manuscript PDF file). The data should be provided as part of the manuscript or its supporting information, or deposited to a public repository. For example, in addition to summary statistics, the data points behind means, medians and variance measures should be available. If there are restrictions on publicly sharing data—e.g. participant privacy or use of data from a third party—those must be specified.

Reviewer #1: Yes

 🡪 Thank you for the comment of reviewer #1.

4. Is the manuscript presented in an intelligible fashion and written in standard English?

PLOS ONE does not copyedit accepted manuscripts, so the language in submitted articles must be clear, correct, and unambiguous. Any typographical or grammatical errors should be corrected at revision, so please note any specific errors here.

Reviewer #1: Yes

🡪 Thank you for the comment of reviewer #1.

5**. Review Comments to the Author**

Please use the space provided to explain your answers to the questions above. You may also include additional comments for the author, including concerns about dual publication, research ethics, or publication ethics. (Please upload your review as an attachment if it exceeds 20,000 characters)

**Reviewer #1:** The paper is well structured, with new interesting information about intraspecific variability of a Chironomid species of high practical interest, living in tap waters.

🡪 Thank you for the comment of reviewer #1.

Only minor adjustments are needed: e. g. Fig. (abbreviated) and Figure (not abbreviated) citations are mixed; at row 257 a t before P. grimmi must be deleted

🡪Lines 201 and 217, we have revised as the format of “Fig. (abbreviated)” citations in the revised manuscript.

🡪Line 264, we have deleted and revised the typo error in the revised manuscript.

6. PLOS authors have the option to publish the peer review history of their article ([what does this mean?](https://track.editorialmanager.com/CL0/https:%2F%2Fjournals.plos.org%2Fplosone%2Fs%2Feditorial-and-peer-review-process%23loc-peer-review-history/1/010f0192c49e8dc7-30b1bdc1-4be1-4abe-9718-1af2ec0c4ea7-000000/U8EgEVIQY0D3VliXXI_zLCsKsROnxNVnd7Y3HWRNygk=182)). If published, this will include your full peer review and any attached files.


If you choose “no”, your identity will remain anonymous but your review may still be made public.


**Do you want your identity to be public for this peer review?** For information about this choice, including consent withdrawal, please see our [Privacy Policy](https://track.editorialmanager.com/CL0/https:%2F%2Fwww.plos.org%2Fprivacy-policy/1/010f0192c49e8dc7-30b1bdc1-4be1-4abe-9718-1af2ec0c4ea7-000000/ul3Q46spFoDEUuxADJ4u9VduXvsgSPfGIZMDXD8Qlbo=182).

Reviewer #1: **Yes:** Bruno Rossaro

 🡪 Thank you for the comment of reviewer Dr. Bruno Rossaro.

**Additional Editor Comments:**

This is an interesting submission, but needs to address to major concerns before being considered for publication. 1) Ptny. grimmii is not a obligate parthenogenetic species, but a cyclical one, perhaps by thermo-dependent regulator, the mechanism is not clear now. At certain condition, some male can be produced. 2) Either adults or larva, chironomid is not a pest insect, but a nuisance insect, casting limited effluence on public health. If these issues can be addressed, I feel the work would be improved and then likely suitable for publication.

🡪We agree with the editor’s comments. The chironomid *P. grimmii* is not an obligate parthenogenetic species and a pest insect.

🡪We have revised the issue in the revised manuscript.

Lines 2-4, we have revised the title as the below.

Before: First report of a major management target species, parthenogenetic chironomid *Paratanytarsus grimmii* (Diptera: Chironomidae) larvae, in drinking water treatment plants (DWTPs) in South Korea

After revision: First report of a major management target species, chironomid *Paratanytarsus grimmii* (Diptera: Chironomidae) larvae, in drinking water treatment plants (DWTPs) in South Korea

Lines 25, 39, 79, 81, 93, 155, 240, 244, We have deleted the word “pest” in the revised manuscript.

Line 27, we have revised the word from “risk” to “possibility” in the revised manuscript.

Lines 39-40, we have revised the word from “pest” to “key management target” in the revised manuscript.

Line 79, we have deleted the “and an apomictic pest” in the revised manuscript.

Line 240, we have revised the word from “seriously” to “negatively” in the revised manuscript.

**Journal requirements:**

When submitting your revision, we need you to address these additional requirements.

1. Please ensure that your manuscript meets PLOS ONE's style requirements, including those for file naming. The PLOS ONE style templates can be found at

<https://journals.plos.org/plosone/s/file?id=wjVg/PLOSOne_formatting_sample_main_body.pdf> and

<https://journals.plos.org/plosone/s/file?id=ba62/PLOSOne_formatting_sample_title_authors_affiliations.pdf>

🡪We have revised the revised manuscript on the PLOS ONE style templates.

2. Thank you for stating the following financial disclosure:

“This work was supported by a grant from the National Institute of Biological Resources (NIBR), funded by the Ministry of Environment (MOE) of the Republic of Korea (NIBR202220202) and the National Research Foundation of Korea, South Korea, funded by the Korean Government (NRF-2018-R1A6A1A-03024314), as well as the Korean Environment Industry & Technology Institute (KEITI) through the Aquatic Ecosystem Conservation Research Program funded by the Korean Ministry of Environment (MOE) (2021003050001).”

Please state what role the funders took in the study.  If the funders had no role, please state: "The funders had no role in study design, data collection and analysis, decision to publish, or preparation of the manuscript."

If this statement is not correct you must amend it as needed.

Please include this amended Role of Funder statement in your cover letter; we will change the online submission form on your behalf.

🡪We have stated as the follow: “The funders had no role in study design, data collection and analysis, decision to publish, or preparation of the manuscript."

3. Thank you for stating the following in the Acknowledgments Section of your manuscript:

“This work was supported by a grant from the National Institute of Biological Resources (NIBR), funded by the Ministry of Environment (MOE) of the Republic of Korea (NIBR202220202) and the National Research Foundation of Korea, South Korea, funded by the Korean Government (NRF-2018-R1A6A1A-03024314), as well as the Korean Environment Industry & Technology Institute (KEITI) through the Aquatic Ecosystem Conservation Research Program funded by the Korean Ministry of Environment (MOE) (2021003050001).”

We note that you have provided funding information that is currently declared in your Funding Statement. However, funding information should not appear in the Acknowledgments section or other areas of your manuscript. We will only publish funding information present in the Funding Statement section of the online submission form.

Please remove any funding-related text from the manuscript and let us know how you would like to update your Funding Statement. Currently, your Funding Statement reads as follows:

“This work was supported by a grant from the National Institute of Biological Resources (NIBR), funded by the Ministry of Environment (MOE) of the Republic of Korea (NIBR202220202) and the National Research Foundation of Korea, South Korea, funded by the Korean Government (NRF-2018-R1A6A1A-03024314), as well as the Korean Environment Industry & Technology Institute (KEITI) through the Aquatic Ecosystem Conservation Research Program funded by the Korean Ministry of Environment (MOE) (2021003050001).”

Please include your amended statements within your cover letter; we will change the online submission form on your behalf.

🡪We have deleted the funding statement in the Acknowledgments section or other areas of the revised manuscript (lines 301-302).

Funding Statement on the online sunmission:

“This work was supported by a grant from the National Institute of Biological Resources (NIBR), funded by the Ministry of Environment (MOE) of the Republic of Korea (NIBR202220202) and the National Research Foundation of Korea, South Korea, funded by the Korean Government (NRF-2018-R1A6A1A-03024314), as well as the Korean Environment Industry & Technology Institute (KEITI) through the Aquatic Ecosystem Conservation Research Program funded by the Korean Ministry of Environment (MOE) (2021003050001).”

4. We note that your Data Availability Statement is currently as follows: [All relevant data are within the manuscript and its Supporting Information files.]

Please confirm at this time whether or not your submission contains all raw data required to replicate the results of your study. Authors must share the “minimal data set” for their submission. PLOS defines the minimal data set to consist of the data required to replicate all study findings reported in the article, as well as related metadata and methods (<https://journals.plos.org/plosone/s/data-availability#loc-minimal-data-set-definition>).

For example, authors should submit the following data:

- The values behind the means, standard deviations and other measures reported;

- The values used to build graphs;

- The points extracted from images for analysis.

Authors do not need to submit their entire data set if only a portion of the data was used in the reported study.

If your submission does not contain these data, please either upload them as Supporting Information files or deposit them to a stable, public repository and provide us with the relevant URLs, DOIs, or accession numbers. For a list of recommended repositories, please see <https://journals.plos.org/plosone/s/recommended-repositories>.

If there are ethical or legal restrictions on sharing a de-identified data set, please explain them in detail (e.g., data contain potentially sensitive information, data are owned by a third-party organization, etc.) and who has imposed them (e.g., an ethics committee). Please also provide contact information for a data access committee, ethics committee, or other institutional body to which data requests may be sent. If data are owned by a third party, please indicate how others may request data access.

🡪We upload the raw excel file for figure 2 as supporting information.

5. We note that Figure 1 in your submission contain [map/satellite] images which may be copyrighted. All PLOS content is published under the Creative Commons Attribution License (CC BY 4.0), which means that the manuscript, images, and Supporting Information files will be freely available online, and any third party is permitted to access, download, copy, distribute, and use these materials in any way, even commercially, with proper attribution. For these reasons, we cannot publish previously copyrighted maps or satellite images created using proprietary data, such as Google software (Google Maps, Street View, and Earth). For more information, see our copyright guidelines: <http://journals.plos.org/plosone/s/licenses-and-copyright>.

We require you to either (1) present written permission from the copyright holder to publish these figures specifically under the CC BY 4.0 license, or (2) remove the figures from your submission:

a. You may seek permission from the original copyright holder of Figure 1 to publish the content specifically under the CC BY 4.0 license.

We recommend that you contact the original copyright holder with the Content Permission Form (<http://journals.plos.org/plosone/s/file?id=7c09/content-permission-form.pdf>) and the following text:

“I request permission for the open-access journal PLOS ONE to publish XXX under the Creative Commons Attribution License (CCAL) CC BY 4.0 (<http://creativecommons.org/licenses/by/4.0/>). Please be aware that this license allows unrestricted use and distribution, even commercially, by third parties. Please reply and provide explicit written permission to publish XXX under a CC BY license and complete the attached form.”

Please upload the completed Content Permission Form or other proof of granted permissions as an ""Other"" file with your submission.

In the figure caption of the copyrighted figure, please include the following text: “Reprinted from [ref] under a CC BY license, with permission from [name of publisher], original copyright [original copyright year].”

b. If you are unable to obtain permission from the original copyright holder to publish these figures under the CC BY 4.0 license or if the copyright holder’s requirements are incompatible with the CC BY 4.0 license, please either i) remove the figure or ii) supply a replacement figure that complies with the CC BY 4.0 license. Please check copyright information on all replacement figures and update the figure caption with source information. If applicable, please specify in the figure caption text when a figure is similar but not identical to the original image and is therefore for illustrative purposes only.

The following resources for replacing copyrighted map figures may be helpful:

USGS National Map Viewer (public domain): <http://viewer.nationalmap.gov/viewer/>

The Gateway to Astronaut Photography of Earth (public domain): <http://eol.jsc.nasa.gov/sseop/clickmap/>

Maps at the CIA (public domain): <https://www.cia.gov/library/publications/the-world-factbook/index.html> and <https://www.cia.gov/library/publications/cia-maps-publications/index.html>

NASA Earth Observatory (public domain): <http://earthobservatory.nasa.gov/>

Landsat: <http://landsat.visibleearth.nasa.gov/>

USGS EROS (Earth Resources Observatory and Science (EROS) Center) (public domain): [http://eros.usgs.gov/#](http://eros.usgs.gov/)

Natural Earth (public domain): <http://www.naturalearthdata.com/>

🡪Fig. 1 is a map image created by the author, so the copyright belongs to the author and permission can be granted under the authority (CC BY 4.0) of the PLOS ONE journal.

Fig. 1 is not previously copyrighted maps or satellite images created using proprietary data, such as Google software.

Thanks in advance.

Yours sincerely,

Ihn-Sil Kwak, Ph.D.

Dept. of Ocean Integrated Science,

Chonnam National University, Chonnam, 550-749, Korea.

E-mail address: [iskwak@chonnam.ac.kr/inkwak@hotmail.com](mailto:iskwak@chonnam.ac.kr/inkwak@hotmail.com)
